# Present Status of Thoracic Surgery

**Published:** 1892-12

**Authors:** 


### Editorial.

### PRESENT STATUS OF THORACIC SURGERY.

Those who have kept abreast of the advances in the surgical resources for chest troubles, as reported in the issues of the Annual of Universal Medical Sciences during the past three years, will fully appreciate the aggressive work in this department. But unfortunately, there are comparatively few of the members of the medical profession who have taken the trouble to ascertain the facts in regard to the various measures which have been adopted in wounds of the thorax or in the modifications of the pleural contents resulting from disease.

Notwithstanding the weight of experience in favor of hermetical sealing of penetrating wounds of the thorax, a new departure has recently been proposed by Dr. Axford, in a paper laid before the Chicago Academy of Medicine, urging a free incision and removal of the blood in traumatism of the chest. In the discussion of Axford's paper, entitled "A Plea for the Lung," the remarks of G. F. Lydston, O. L. Schmidt, J. G. Kiernan and H. N. Moyer, of Chicago, Ill., present some points of practical importance. It is a peculiar fact, says the first named gentleman, that the lung has received less attention in modern surgical procedures than other important organs. The chief step in advance, that has been taken, is the conservative one of the establishment of the principle to avoid probing in lung gun-shot wounds. If a foreign body should be located and removed, such as a bullet, the removal would be attended with more damage than the bullet would if left alone.

The older surgeons frequently bled from the arm to the point of syncope in the hope that the rest secured for the circulation would permit the formation of a clot at the site of the internal injury. This principle of rest is mostly the foundation upon which lung surgery should be based. The foreign body being left to take care of itself, the menacing indications are to check hemorrhages, to secure asepsis of the wound and to promote rest.

Many of the wounds of the lung do very well if left alone. Everything depends upon whether blood vessels of any size have been cut across. The power of absorption by serous membranes is often forgotton. The serous sacs are practically huge lymph sacs and absorption from their surfaces is very active.

As Dr. Axford has suggested, it is impossible to get the pleural cavity into an aseptic condition when blood mixed air is present in considerable quantity. His suggestion of a free incision and the removal of the blood is, it seems to Lydston, logical—not as applied to every case, but to the exceptional cases, such as that upon which he has based his paper.

As the pleural cavity is already opened, he sees no objection to its free incision in suitable cases. He thinks it would be better to operate as a primary procedure for the checking of hemorrhage and the avoidance of sepsis, than to wait until a large accumulation of blood has occurred and septic decomposition has already taken place.

Schmidt points out that free incisions into the pleural cavity would convert this into an open pneumothorax, having external openings, and in all probability, also a communication with a bronchus, for only in case of a wound of the lung is this measure to be praticed. After the removal of the blood from the cavity, the favorable influence of the pressure of the blood previously in the cavity would be lost and a cessation of hemorrhage would then be due to (1) retraction of the lung tissue produced by its elasticity, producing compression of the bleeding vessel, (2) formation of a clot, due to exsanquination, (3) less mobility of the lung during respiration. Then these causes would act together; and, inasmuch as the 1st and 3rd are very indefinite in their hemostatic influence, the second cause would be the one most usually effective. But stoppage of hemorrhage by copious loss of blood is very unreliable and is a principle never used unless more certain means cannot be brought into play in the case.

Kiernan said, in dealing with the question raised by Axford, that lung surgery had received attention ere the days of antiseptics and anæsthetics. In the Hunterian museum are the lungs of a man which had been transfixed by a jig-shaft in 1802, from which he recovered. An English sailor in 1837 was pinned to the deck of a ship by the pivot of a try-sail, which pressed through the chest, and yet he recovered. Hale of Minersville, Pa., reports a case of a 25 year old man, who had a stab wound 1 1-4 in. long in the left side, from which a portion of the lung protruded. This was excised measuring six inches long, two and one half inches in diameter at greatest width, and one inch where it was excised. The patient made a good recovery. It is the conviction of Kiernan that under antisepsis and anæsthesia the chief risks, shock and blood-poisoning, could be reduced. A patient was operated on by Fenger, ten years ago, for a gangrene cavity of the thorax who was in excellent health despite a careless mode of life.

Moyer states that one of the problems incident to the question raised by Dr. Axford is a medico-legal one. If death results from the operation on a victim of a wound, the question of how far can this be charged to the operation, might become of serious importance. Willard is of opinion that entrance of air into the pleural cavity is far more serious as regards lung collapse, when the pulmonary tissues were normal than when they were diseased. He states, and his experience is corroborated by Fenger, that incisions into tuberculous, gangrenous or suppurating pulmonary tissue can be safely undertaken. Better results are therefore to be expected in pulmonary gangrene and abscess, than from any other method of treatment.

Dr. Axford, in concluding the discussion, said he would not think of operating in all cases, only those where there was immediate danger of large hemorrhage and subsequent danger from sepsis by decomposition of blood mixed with air.—(*The Medical Standard*, February and April, 1892).

It would appear from the favorable views expressed by some of the speakers in regard to the proposition of Dr. Axford, to lay open the chest in cases of considerable hemorrhage into the pleural cavity, that this procedure met the sanction of surgeons at the present day. But on the contrary, this practice is in opposition to the best established principles which govern the treatment of penetrating wounds of the thorax; and an entirely different mode of dealing with this class of cases has been adopted by those surgeons having the largest experience in thoracic wounds. It is now the recognized course of treatment to allow of the escape of all the blood which may flow out of the wound when placed in a dependent position and afterwards close the opening or openings into the pleural cavity, by suture, so as to effectually shut off the thoracic cavity from the external air. This secures the most satisfactory results.

J. McF. G.

